# A Lightweight Frozen Multi-Convolution Dual-Branch Network for Efficient sEMG-Based Gesture Recognition

**DOI:** 10.3390/s26020580

**Published:** 2026-01-15

**Authors:** Shengbiao Wu, Zhezhe Lv, Yuehong Li, Chengmin Fang, Tao You, Jiazheng Gui

**Affiliations:** 1 School of Electronics and Electrical Engineering, East China University of Technology, Nanchang 330013, China; 2024110326@ecut.edu.cn (Z.L.);; 2 Jiangxi Industry Technology Research Institute of Rehabilitation Assistance, Nanchang 330013, China

**Keywords:** lightweight neural network, surface electromyography (sEMG), gesture recognition, random convolution

## Abstract

Gesture recognition is important for rehabilitation assistance and intelligent prosthetic control. However, surface electromyography (sEMG) signals exhibit strong non-stationarity, and conventional deep-learning models require long training time and high computational cost, limiting their use on resource-constrained devices. This study proposes a Frozen Multi-Convolution Dual-Branch Network (FMC-DBNet) to address these challenges. The model employs randomly initialized and fixed convolutional kernels for training-free multi-scale feature extraction, substantially reducing computational overhead. A dual-branch architecture is adopted to capture complementary temporal and physiological patterns from raw sEMG signals and intrinsic mode functions (IMFs) obtained through variational mode decomposition (VMD). In addition, positive-proportion (PPV) and global-average-pooling (GAP) statistics enhance lightweight multi-resolution representation. Experiments on the Ninapro DB1 dataset show that FMC-DBNet achieves an average accuracy of 96.4% ± 1.9% across 27 subjects and reduces training time by approximately 90% compared with a conventional trainable CNN baseline. These results demonstrate that frozen random-convolution structures provide an efficient and robust alternative to fully trained deep networks, offering a promising solution for low-power and computationally efficient sEMG gesture recognition.

## 1. Introduction

Gesture recognition plays a crucial role in intelligent prosthetic control and rehabilitation assistance, serving as a key pathway toward natural human–machine interaction [1,2,3]. Surface electromyography (sEMG), a non-invasive signal reflecting neuromuscular activity, has been widely applied in upper-limb rehabilitation, prosthetic control, and human–machine interface research [4]. Despite recent advances in sensor technology and computational platforms, sEMG-based gesture recognition remains challenging due to its strong non-stationarity, substantial inter-subject variability, and fluctuations across repetitions and muscle conditions [5,6]. These characteristics complicate the design of models that are both robust and computationally efficient, particularly when targeting embedded or low-power systems with limited resources.

Traditional sEMG-based gesture recognition methods primarily rely on handcrafted features in the time, frequency, and time–frequency domains, combined with classifiers such as support vector machines (SVMs) and hidden Markov models (HMMs) [7,8,9,10]. Although such approaches are computationally lightweight, manually designed features struggle to capture the nonlinear, transient, and task-dependent properties of sEMG signals, which show pronounced non-stationary and subject-specific behaviors [11,12,13,14,15]. As a result, these systems often require careful feature engineering and suffer from performance degradation when the recording conditions, subjects, or tasks deviate from those seen during design.

Deep learning techniques have alleviated many of these limitations by enabling end-to-end feature learning directly from raw or minimally processed sEMG data [16,17,18,19]. Convolutional neural networks (CNNs) have become the dominant paradigm in recent years, owing to their ability to learn hierarchical temporal–spatial representations [20,21]. On this basis, a variety of architectures have been proposed, including CNN–LSTM hybrids for dynamic gesture recognition [22], CNN models constructed on Gramian angular field representations of sEMG [23], and dual-branch CNNs that integrate global and local cues [24]. Multi-scale convolutions and attention mechanisms have further enhanced the representational capacity of these networks [25,26]. However, these deep models typically rely on a large number of trainable parameters and backpropagation-based optimization, leading to high computational cost and long training time, which limit their deployment on low-power or resource-constrained devices.

To reduce training overhead while retaining strong discriminative power, researchers have started to explore random-feature-based learning paradigms. Random vector functional link (RVFL) networks employ fixed random weights in the hidden layer to project inputs into a high-dimensional feature space, after which only a linear readout needs to be trained [27,28,29]. The ROCKET method extends this idea to the convolutional domain by applying fixed random convolutional kernels to capture multi-scale temporal patterns, achieving classification performance comparable to that of fully trainable CNNs without backpropagation [30,31,32,33]. Related work on convolutional RVFL (CRVFL) architectures further demonstrates the feasibility of random convolutional structures for image and physiological signal processing [34,35]. Nevertheless, most existing random-feature or random-convolution approaches are designed for single-source or single-channel inputs and lack mechanisms to jointly model multiple physiological components of sEMG, such as the raw signals and intrinsic mode functions (IMFs) obtained via variational mode decomposition (VMD).

To address these limitations, this study follows the efficient learning paradigm of fixed feature extraction with a lightweight classifier and proposes a Frozen Multi-Convolution Dual-Branch Network (FMC-DBNet) for sEMG-based gesture recognition. The model employs randomly initialized and fixed convolutional kernels to perform training-free multi-scale feature mapping, substantially reducing computational overhead. A dual-branch architecture is adopted to separately process raw sEMG signals and VMD-derived IMFs, enabling physiological-level signal decoupling and complementary modeling of neuromuscular activity. Furthermore, by integrating multi-scale convolutions with positive-proportion (PPV) and global-average-pooling (GAP) statistics, the proposed network constructs lightweight multi-resolution representations of both temporal and physiological patterns. Experiments on the Ninapro DB1 dataset demonstrate that the proposed FMC-DBNet can markedly reduce training time while maintaining high recognition accuracy, indicating that frozen random-convolution structures offer a practical and effective solution for efficient sEMG gesture recognition.

## 2. Materials and Methods

**Figure 1:**
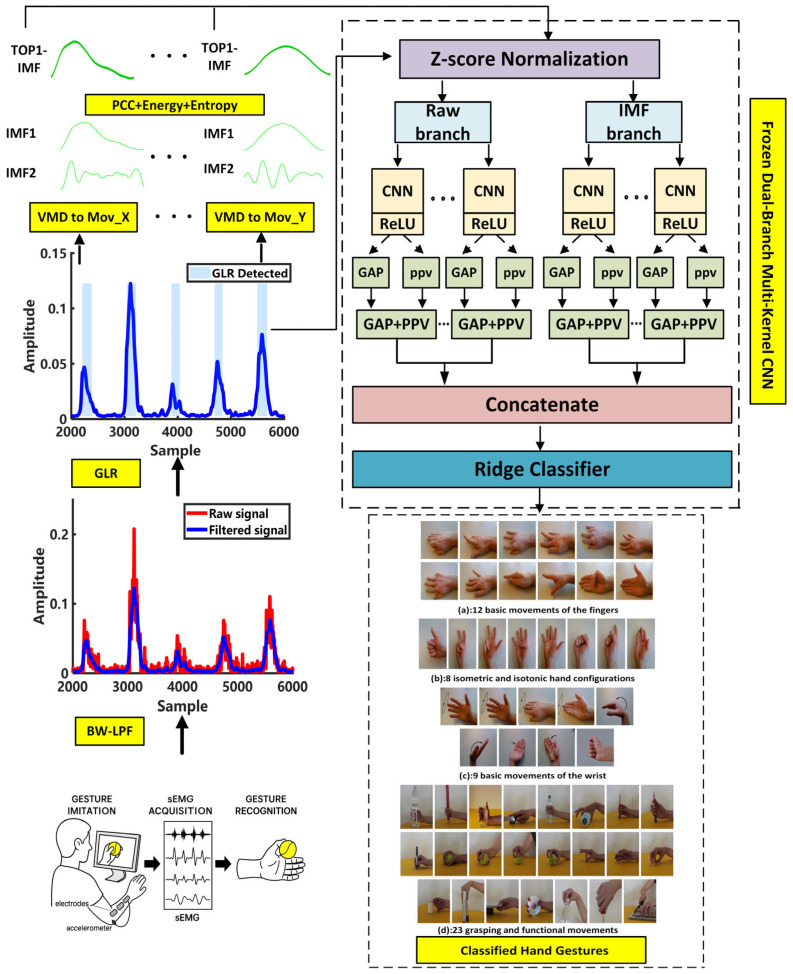
Schematic diagram of the proposed FMC-DBNet gesture recognition system. The framework includes signal acquisition, preprocessing, motion-segment detection, VMD-based feature decomposition, and dual-branch feature extraction followed by Ridge classification.

Figure 1 illustrates the overall framework of the proposed Frozen Multi-Convolution Dual-Branch Network (FMC-DBNet) for sEMG-based gesture recognition. The system consists of four main stages: (1) signal preprocessing and motion-segment detection; (2) variational mode decomposition (VMD) and IMF selection; (3) dual-branch feature extraction using fixed multi-scale convolutions; and (4) feature fusion and linear discrimination. The following subsections provide detailed descriptions of the design and implementation of each stage.

### 2.1. NinaPro Dataset

This study employs the DB1 dataset from the publicly available NinaPro database [36]. NinaPro is a multimodal open-access repository designed to support research on electromyography and prosthetic control by providing standardized data resources for gesture-recognition studies. As one of its primary subsets, DB1 contains a diverse set of gesture classes, a sufficiently large subject cohort, and rigorously standardized acquisition and annotation procedures. These characteristics make DB1 a widely recognized benchmark for sEMG-based gesture recognition, enabling fair comparison, reproducibility, and scientifically reliable model evaluation.

DB1 includes sEMG recordings from 27 subjects (20 males, 7 females; average age ≈ 28 years). Each subject performs 52 gestures (including rest), covering finger motions, static and dynamic gestures, wrist movements, and grasping actions. Signals are recorded using ten Otto Bock MyoBock 13E200 (Otto Bock HealthCare GmbH, Duderstadt, Germany) surface electrodes at a 100 Hz sampling rate, providing stable forearm muscle-activity measurements for subsequent analysis.

A within-subject evaluation protocol is adopted. For each subject, repetitions {2, 5, 10} are used for testing, while {1, 3, 4, 6, 7, 8, 9} serve as the training set, following commonly used partitioning strategies in prior work [37,38,39]. Model performance is assessed using five standard metrics: accuracy (ACC), precision (PRE), recall (REC), F1-score (F1-S), and Matthews correlation coefficient (MCC). Definitions of these metrics are provided in Table 1.

### 2.2. Signal Preprocessing

The raw sEMG signals are affected by intrinsic and external noise and therefore require systematic preprocessing. Following the Ninapro DB1 acquisition and classification protocol, the signals are first subject to interference suppression at the acquisition stage. Subsequently, a first-order Butterworth low-pass filter with a cutoff frequency of 1 Hz is applied prior to classification to attenuate high-frequency fluctuations. Rest segments are removed, and only motion segments are retained for subsequent analysis [36].

The motion labels provided in DB1 are obtained by visual inspection, and their onset/offset boundaries may contain inaccuracies caused by reaction delays or early terminations. Such boundary deviations can lead to incomplete motion segments or mislabeled rest intervals, which may hinder the learning of discriminative gesture patterns.

To mitigate this issue, an automatic motion-boundary refinement method is employed, which combines multi-channel generalized likelihood ratio (GLR)-based change-point detection with a voting-based fusion strategy. Specifically, for each annotated segment, the temporal window is expanded to include surrounding context, and each channel is modeled under a two-state statistical hypothesis corresponding to rest and motion. The motion onset and offset are estimated using a likelihood-based criterion within the expanded window.

**Figure 2:**
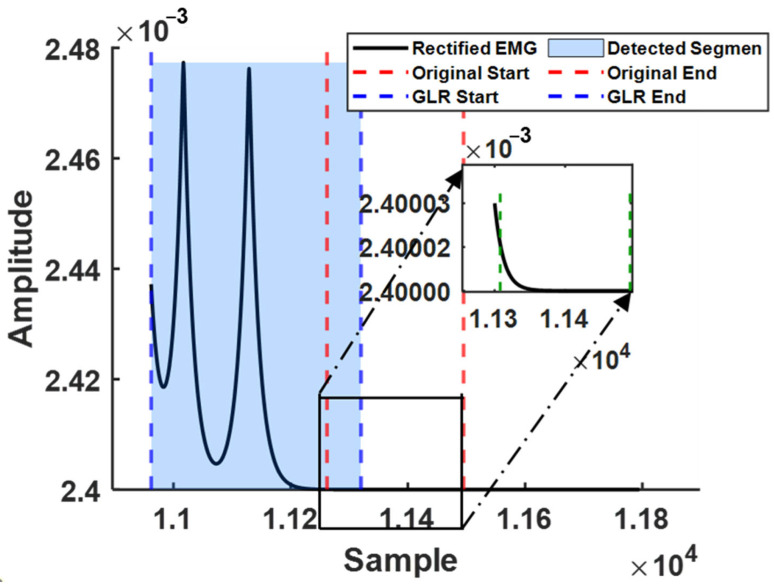
The solid black line denotes the rectified sEMG signal, while blue dashed lines mark the start and end boundaries detected by the GLR method. Red dashed lines represent the original label boundaries, and the light-blue shaded area indicates the detected active segment. The inset in the upper right shows a zoomed-in view of the labeled segment, illustrating the boundary deviation between the original label and the actual waveform.

To improve robustness against channel-specific noise, a voting fusion is applied across channels, where the earliest detected onset and the latest detected offset are selected as the final global boundaries. This procedure enables consistent boundary refinement across subjects and repetitions. Figure 2 illustrates a representative example (Subject S1, Channel 6, Gesture 2, Repetition 4), in which the refined segment exhibits a more complete and continuous motion pattern, demonstrating the effectiveness of the proposed boundary-refinement approach.

### 2.3. Variational Mode Decomposition (VMD)

sEMG signals acquired during gesture execution exhibit pronounced nonlinearity and non-stationarity. To effectively separate frequency components and suppress non-stationary noise, this study adopts an improved variational mode decomposition (VMD) method. Unlike empirical mode decomposition (EMD) and wavelet-based approaches, VMD is formulated within a strict variational optimization framework, achieving a more favorable balance between time–frequency localization and decomposition stability [40].

VMD decomposes a signal into a set of bandwidth-limited intrinsic mode functions (IMFs) by minimizing the sum of their individual bandwidths. In Algorithm 1, $\hat{f}\left(\omega \right)\;$denotes the one-sided analytic spectrum of the input sEMG segment; ${\hat{u}}_{k}^{\left(\alpha \right)}(\omega )$ is the frequency-domain representation of the k-th IMF at iteration α; $\omega_{k}^{\left(\alpha \right)}$ is its center frequency; and $\lambda^{\left(\alpha \right)}(\omega )$ is the Lagrange multiplier enforcing reconstruction consistency. The algorithm employs the alternating direction method of multipliers (ADMM) to iteratively update each IMF and its corresponding center frequency until convergence. The key parameters are: number of modes K=2, bandwidth penalty α=2000, dual-ascent step τ=0, and convergence tolerance $1\times 10^{-7}$. Each iteration updates the IMFs, adjusts center frequencies using spectral centroids, and then updates the multiplier to accelerate convergence.

To improve the practical applicability of VMD, three refinements are incorporated:

- DC-component correction, which mitigates baseline drift;
- Amplitude calibration using full-length least-squares normalization to ensure energy consistency;
- Quality indicators, including reconstruction error, energy ratio, and fallback rate.

Experimental results show that the median reconstruction error across all subjects ranges from 0.9% to 1.6%, with the 95th percentile below 10%. The IMF energy ratio remains stable at approximately 0.50, demonstrating numerically stable and highly reproducible decomposition. The resulting IMFs effectively suppress low-frequency drift and non-stationary noise while preserving the original time–frequency structure, thereby providing more robust inputs for subsequent feature extraction and gesture recognition. The sensitivity of the VMD parameters, particularly the number of modes K and the bandwidth penalty factor α, is systematically analyzed in the experimental section to assess the robustness of the adopted configuration.
**Algorithm 1:** sEMG signal decomposition using improved VMDInitialize ${\hat{u}}_{k}^{\left(1\right)},\;\omega_{k}^{\left(1\right)},\;\lambda^{\left(1\right)}(\omega ),\;a\leftarrow 0$**repeat** a←a+1 **for** k = 1: K **do** Update each IMF in the frequency domain:
${\hat{u}}_{k}^{\left(a+1\right)}(\omega )\leftarrow \frac{\hat{f}(\omega )-\sum_{j\neq k}{\hat{u}}_{j}^{\left(a\right)}(\omega )+\frac{1}{2}\lambda^{\left(a\right)}(\omega )\;}{1+2\alpha {\left(\omega -\omega_{k}^{\left(a\right)}\right)}^{2}}$ Update the center frequency:
$\omega_{k}^{\left(a+1\right)}\leftarrow \frac{{\int_{0}^{\infty }\omega \left|{\hat{u}}_{k}^{\left(a+1\right)}(\omega )\right|}^{2}d\omega }{{\int_{0}^{\infty }\left|{\hat{u}}_{k}^{\left(a+1\right)}(\omega )\right|}^{2}d\omega }$ **end for** Update the Lagrange multiplier (dual ascent):
$\lambda^{\left(a+1\right)}(\omega )\leftarrow \lambda^{\left(a\right)}(\omega )+\tau \left(\hat{f}(\omega )-\sum_{k=1}^{K}{\hat{u}}_{k}^{\left(a+1\right)}(\omega )\right)$ **until** convergence: 
$\sum_{k=1}^{K}\frac{{\left\|{\hat{u}}_{k}^{\left(a+1\right)}-{\hat{u}}_{k}^{\left(a\right)}\right\|}_{2}^{2}}{{\left\|{\hat{u}}_{k}^{\left(a\right)}\right\|}_{2}^{2}}<\mathrm{tol}$


### 2.4. IMF Selection Based on Weighted Feature Indicators

After VMD decomposition, each sEMG segment is decomposed into several intrinsic mode functions (IMFs). Because some IMFs mainly contain noise or weakly correlated components, relying on a single selection criterion may overlook important structural characteristics of the signal [41]. To address this issue, we adopt a weighted multi-feature evaluation strategy to automatically identify the most discriminative IMFs.

High-quality IMFs generally exhibit three properties: (1) a high energy ratio, indicating a dominant contribution to the signal; (2) strong correlation with the original sEMG, preserving major motion-related information; and (3) low complexity, reflecting stable structural behavior. Accordingly, three complementary indicators—energy ratio $E_{i}$, Pearson correlation coefficient $\rho_{i}$, and sample entropy$\;S_{i}$—are used. All indicators are normalized using the min–max method:

(1) $$E\prime_{i}=\frac{E_{i}-\min\left(E\right)}{\max\left(E\right)-\min\left(E\right)},$$

(2) $$\rho \prime_{i}=\frac{\left|\rho_{i}\right|-\min\left(\left|\rho \right|\right)}{\max\left(\left|\rho \right|\right)-\min\left(\left|\rho \right|\right)},$$

(3) $$S\prime_{i}=\frac{S_{i}-\min\left(S\right)}{\max\left(S\right)-\min\left(S\right)}.$$

The overall IMF score is then defined as:

(4) $$Score_{i}=\omega_{p}{\rho^{\prime }}_{i}+\omega_{e}E_{i}+\omega_{s}(1-{S^{\prime }}_{i}),$$

where the weights $\omega_{p}=0.4$, $\omega_{e}=0.4$, and $\omega_{s}=0.2$ are determined following established multi-criteria evaluation principles and validated through extensive cross-validation. For each channel, IMFs are ranked in descending order of ${Score}_{i}$, and the top-N scoring modes are retained for subsequent feature extraction and modeling.

**Figure 3:**
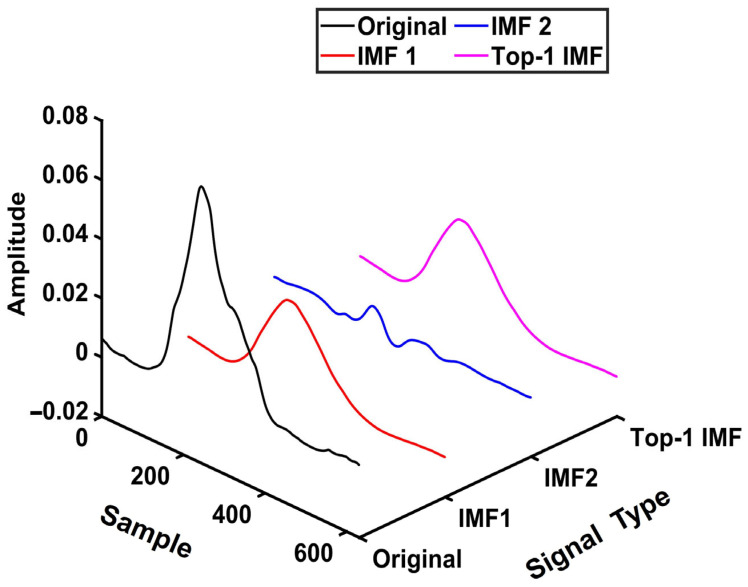
Three-dimensional waveform comparison of the original sEMG signal and its VMD decomposition results (IMF1 and IMF2) under K = 2, together with the Top-1 IMF selected based on the weighted feature indicators.

Table 2 shows that, for Subject S1 (Gesture 5, Repetition 8, Channel 1), IMF1 achieves higher correlation, higher energy ratio, and lower entropy than IMF2, resulting in the highest overall score and being selected as the effective IMF. Figure 3 presents the three-dimensional waveform comparison among the raw signal, decomposed IMFs, and the selected top-1 IMF, illustrating the fidelity and representativeness of the selected mode.

To ensure consistent input dimensions, all motion segments are standardized to a fixed window length of 1100 samples. Segments shorter than this length are zero-padded, whereas longer segments are truncated according to the statistical distribution of valid segments in the Ninapro DB1 dataset.

### 2.5. Model Architecture

#### 2.5.1. Theoretical Motivation

Convolutional neural networks (CNNs) have been widely applied in time-series analysis owing to their ability to automatically extract local temporal features without handcrafted design [42]. In addition to the conventional spatial interpretation, recent studies provide a frequency-domain perspective, showing that convolutional kernels function as tunable filters capable of capturing multi-scale spectral structures in the input signal [43,44,45].

Saxe et al. further demonstrated that even randomly initialized kernels exhibit inherent frequency selectivity and naturally respond to specific spectral components without any training [46]. This phenomenon forms the theoretical basis of Random CNNs, in which fixed random filters combined with lightweight linear classifiers enable highly efficient feature extraction.

Motivated by this paradigm, the proposed Frozen Multi-Convolution Dual-Branch Network (FMC-DBNet) employs multi-scale random convolutions to achieve stable, training-free feature mapping. The dual-branch architecture further enhances physiological representation by jointly modeling raw sEMG signals and VMD-derived components.

#### 2.5.2. Overall Model Architecture

The FMC-DBNet consists of two frozen random convolutional feature extraction branches and a linear classification module, as illustrated in Figure 1. After standardization, the input signals are fed separately into the raw signal branch (Raw Branch) and the VMD component branch (IMF Branch). The two branches share an identical architecture but do not share parameters. Each branch contains multiple one-dimensional random convolutional layers, where different combinations of kernel sizes and dilation rates are used to capture multi-scale temporal characteristics. The convolution kernels remain frozen after random initialization and are not updated through backpropagation.

The convolution operation is defined as follows:

(5) $$h(i,j)=f\left(b+\sum_{c=1}^{C}\left(k\left(j,c\right)\times x(i,c)\right)\right),$$

where x(i,c) denotes the input signal at time step i for channel c; k(j,c) represents the convolution kernel; b is the bias term; and f(⋅) denotes the nonlinear activation function (ReLU in this study). The convolutional weights ω are randomly initialized according to:

(6) $$\forall \omega \in W,\;\omega \sim N\left(0,\frac{2}{C_{in}\cdot k}\right),$$

$C_{\mathrm{in}}$ denotes the number of input channels, and k represents the kernel length. A “same” padding strategy is employed, and the corresponding calculation formula is given by:

(7) $$padding=\left[\frac{dilation\cdot (k-1)}{2}\right]$$

This configuration ensures that the temporal length of the feature sequence remains consistent across layers during propagation.

#### 2.5.3. Feature Extraction and Statistical Mapping

To enhance the stability and discriminability of the extracted features, two statistical measures are computed after each convolutional output: Global Average Pooling (GAP) and Proportion of Positive Values (PPV). The former reflects the overall activation strength of each channel, while the latter represents the proportion of positive outputs after ReLU activation. Their definitions are given as follows:

(8) $$GAP_{i}=\frac{1}{T}\sum_{t=1}^{T}z_{i}(t),$$

(9) $$PPV_{i}=\frac{1}{T}\sum_{t=1}^{T}\left[z_{i}(t)>0\right],$$

where T denotes the temporal length, and $\;z_{i}\left(t\right)$ represents the activation value of channel i at time t.

For each convolutional kernel, the GAP and PPV features are concatenated to form a multi-scale subspace representation. Subsequently, the features extracted from the Raw and IMF branches are fused along the channel dimension to construct an integrated multi-scale time–frequency feature vector. This architecture preserves both the global trends of the raw signal and the local details of the VMD components, enabling robust modeling of sEMG signals.

#### 2.5.4. Feature Fusion and Classification

The fused global features are input into a linear discriminant classifier for final recognition. This study employs a Ridge classifier, a linear regression model with an L_2_ regularization term, in which the weights are estimated by minimizing the following objective function:

(10) $$\underset{W}{\min}{\left\|DW-Y\right\|}_{F}^{2}+\alpha {\left\|W\right\|}_{F}^{2}$$

Here, D denotes the feature matrix of the training samples, Y is the one-hot encoded class indicator matrix, and α represents the regularization coefficient. The closed-form solution is given as:

(11) $$W={\left(D^{T}D+\alpha I\right)}^{-1}D^{T}Y$$

During the testing phase, the feature vector $f_{test}$ is linearly projected to obtain class scores, and the predicted label is determined by the index corresponding to the maximum score:

(12) $$\hat{y}=\mathrm{argmax}(f_{test}^{T}W)$$

The Ridge classifier features a simple structure and low computational cost. It achieves high recognition accuracy while avoiding the backpropagation process of deep networks, making it suitable for lightweight scenarios with frozen convolutional feature extractors.

#### 2.5.5. Summary of Model Architecture

In summary, the proposed FMC-DBNet is centered on a dual-branch frozen random convolutional architecture that performs hierarchical representation of sEMG signals through multi-scale convolution and statistical mapping. The model comprises four main components—signal input, feature extraction, feature fusion, and linear classification—providing a complete implementation framework for the subsequent experiments.

## 3. Results

### 3.1. Experimental Setup

All experiments were conducted on a Windows 10 (64-bit) system equipped with an Intel i5-9300H CPU (Intel Corporation, Santa Clara, CA, USA), 16 GB RAM, and an NVIDIA GTX 1650 GPU (NVIDIA Corporation, Santa Clara, CA, USA). The implementation was developed using PyTorch 1.13.1 (CUDA 11.6, cuDNN 8) and scikit-learn 1.6.1, with fixed random seeds to ensure reproducibility.

The experimental pipeline followed Section 2.2, Section 2.3 and Section 2.4, including GLR-based boundary correction, VMD decomposition, IMF selection, and segment standardization. According to the protocol in Section 2.1, repetitions {2, 5, 10} were used for testing, while {1, 3, 4, 6, 7, 8, 9} served as training data. All motion segments were truncated or zero-padded to 1100 samples to ensure consistent input length. The frozen CNN feature extractor remained unchanged, and only the final Ridge classifier was trained. The detailed experimental configuration and implementation settings are summarized in Appendix A.

For each subject, five independent experiments were performed using random seeds {42, 123, 777, 2024, 2025}, and a five-fold cross-validation procedure was applied within each experiment. Training time was defined as the total duration of feature extraction and classifier training, and the reported values represent the mean and standard deviation over five runs. Model performance was evaluated using five metrics—ACC, PRE, REC, F1-S, and MCC—computed on the designated test set.

### 3.2. Frequency-Domain Feature Analysis

To verify whether the sEMG signals used in this study exhibit discriminative characteristics in the frequency domain and to provide theoretical support for the design of multi-scale convolutional kernels, this section conducts a frequency-domain analysis on the training samples. We employed the Welch method (Hamming window, 50% overlap, 512-point FFT) to estimate the power spectral density (PSD) within the 0–5 Hz range and analyzed the spectral distributions associated with different gesture classes.

**Figure 4:**
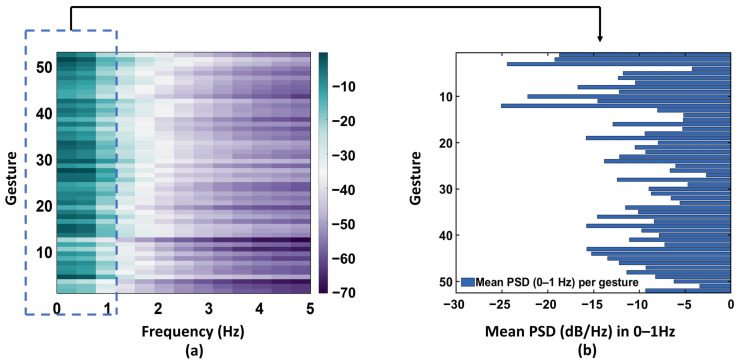
(**a**) PSD heatmap of 52 gestures from Subject S1 in the 0–5 Hz band; (**b**) Mean PSD of all gestures in the 0–1 Hz band. Distinct low-frequency patterns appear across gestures, indicating clear inter-gesture spectral differences.

Figure 4 summarizes the global spectral characteristics of the 52 gesture classes from Subject S1. As shown in Figure 4a, clear low-frequency differences appear across gestures within the 0–5 Hz band, particularly in the 0–1 Hz range. Some gestures exhibit pronounced low-frequency energy peaks, whereas others show relatively flat spectral responses. To further emphasize these distinctions, Figure 4b presents the mean PSD in the 0–1 Hz band for each gesture class, where the largest inter-class variations are observed.

**Figure 5:**
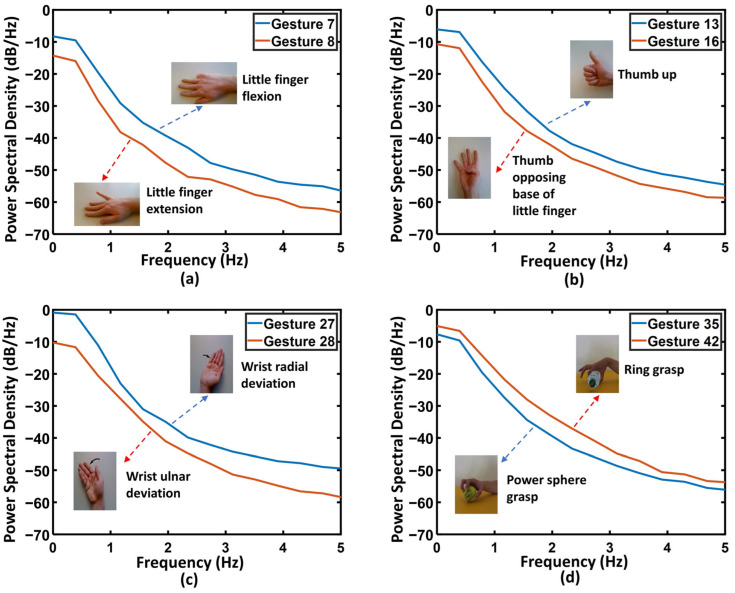
Average PSD comparison (Welch method) of four gesture categories from Subject S1: (**a**) Finger, (**b**) Posture, (**c**) Wrist, and (**d**) Grasping. Differences in the 0–1 Hz band highlight the discriminative spectral features of sEMG.

To examine gesture-specific spectral behavior in more detail, four representative gesture categories—Finger, Posture, Wrist, and Grasping—were selected, and pairwise spectral comparisons were conducted for gestures with similar visual appearances or physiological characteristics (Figure 5). The comparisons show that within the 0–1 Hz band, the energy-decay patterns and spectral shapes differ substantially across gestures—for example, between Little finger flexion and Little finger extension (Figure 5a), and between Thumb up and Thumb opposing (Figure 5b).

Overall, the results indicate that the sEMG signals used in this study contain their most discriminative information within the 0–1 Hz low-frequency range. These findings directly support the multi-scale convolution-kernel design in FMC-DBNet: randomly initialized kernels with different receptive fields can capture distinct frequency components without training, thereby enhancing the network’s ability to model multi-scale temporal structures.

### 3.3. Network Configuration

As shown in Table 3, the proposed FMC-DBNet adopts two structurally symmetric but parameter-independent 1D convolutional branches for processing raw sEMG signals and VMD-derived IMF components.

Although the two branches share the same architecture, their convolutional kernels are not shared, allowing each branch to learn domain-specific and complementary frequency patterns. Each branch contains three parallel convolutional modules whose kernel lengths k and dilation rates d are determined through a Tree-structured Parzen Estimator (TPE) search. The search space was k∈{1,3,5,7,9,11} and d∈{1,3,5,7,9,11}. Using the average five-fold test accuracy across 27 subjects as the objective, 200 optimization rounds yielded the optimal multi-scale configuration (k,d)={(3,1),(5,3),(11,8)}, corresponding respectively to local, mid-range, and long-range temporal receptive fields.

Each convolutional module outputs 256 channels with weights initialized by Kaiming Normal initialization and then frozen to ensure stability and reproducibility. Same padding is used to maintain the input length of 1100 samples, and ReLU activation is applied after convolution. From each module, two statistical descriptors are extracted: global average pooling (GAP), representing mean activation strength, and the proportion of positive values (PPV), reflecting sparsity and activation distribution. Concatenating GAP and PPV results in a 512-dimensional feature vector per module, and combining the three modules produces a 1536-dimensional multi-scale representation for each branch. The raw-signal and IMF branches are then fused into a final 3072-dimensional feature vector.

Since all convolutional parameters remain fixed, an $L_{2}$-regularized Ridge classifier serves as the only trainable component. The regularization coefficient α was optimized within [0.01,1] using TPE with logarithmic sampling, yielding α=0.039. This “frozen convolution + linear readout” design eliminates backpropagation-related overhead, significantly reduces training cost, and improves robustness, while still retaining high recognition performance. Overall, FMC-DBNet combines multi-scale convolutions, dual-branch feature extraction, and statistical aggregation to effectively capture the time–frequency characteristics of sEMG signals without relying on trainable convolution kernels.

### 3.4. Model Training and Evaluation

To evaluate the proposed FMC-DBNet, independent experiments were conducted on 27 subjects from the Ninapro DB1 dataset. All subjects used GLR-corrected motion segments and VMD-selected IMF features as inputs. Throughout training, all convolutional kernels remained frozen, and only the Ridge classifier parameters were optimized. To analyze model robustness, each subject was trained under five random seeds (42, 123, 777, 2024, 2025), with five-fold cross-validation performed in each run.

**Figure 6:**
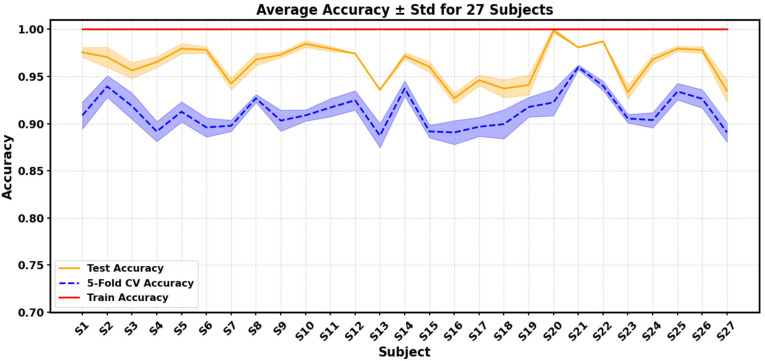
Line-plot visualization of training, five-fold cross-validation, and test accuracies of the proposed FMC-DBNet across 27 subjects. Shaded areas indicate the standard deviation over five independent runs.

As shown in Figure 6, the trends of training, cross-validation, and test accuracy across subjects are highly consistent, with most standard deviations below 2–3%, indicating low sensitivity to initialization randomness and stable performance across repeated runs. A few subjects (e.g., S16 and S20) exhibit slightly lower accuracy, possibly due to weaker sEMG amplitudes or higher noise levels, yet the overall pattern remains consistent.

**Figure 7:**
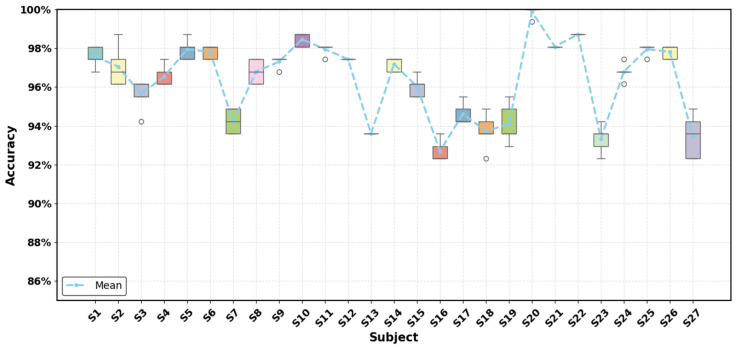
Boxplot of test accuracies for the 27 subjects from the Ninapro DB1 dataset. Each box summarizes the accuracy distribution over five random-seed experiments, with the line, box and whiskers representing the median, interquartile range, and full range, respectively. Hollow circles indicate outliers.

Figure 7 further illustrates the distribution of test accuracy across the five runs for each subject. For most subjects, the boxplots are highly compact with minimal variance, and several subjects even exhibit identical results across all five runs, demonstrating strong robustness to random perturbations.

**Figure 8:**
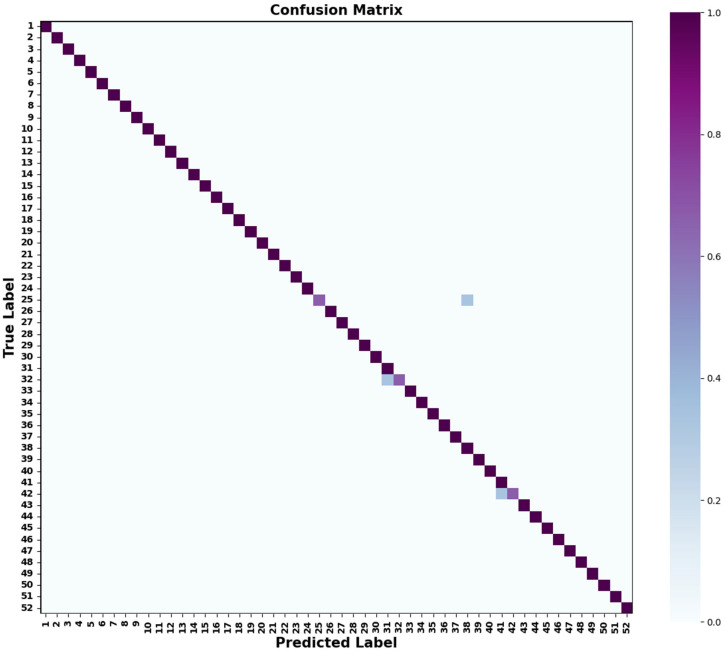
Confusion Matrix of Kinds of Gestures.

To examine class-level performance for a stable subject, Figure 8 provides the 52-class confusion matrix for subject S21.

Most predictions cluster near the diagonal, indicating strong overall recognition capability. Minor confusions occur between a few gesture pairs (e.g., 25/38, 32/31, 42/41), primarily among gestures with similar motion characteristics, suggesting room for enhancing inter-class separability in future work.

Overall, the results demonstrate that the proposed dual-branch multi-convolution frozen CNN model achieves stable, repeatable, and reliable recognition performance across subjects and random initializations, indicating strong potential for practical deployment.

### 3.5. Baseline Model

#### 3.5.1. CNN Baseline

To ensure a fair and controlled comparison with the proposed FMC-DBNet, a conventional trainable single-branch 1D CNN was implemented as the baseline model. This network processes only the raw sEMG signals obtained after GLR-based segmentation and standardized to a fixed length of 1100 samples, without incorporating IMF information or frozen convolutional modules.

The baseline CNN consists of three sequential 1D convolutional layers with channel sizes of 64, 128, and 256 and kernel sizes of 5, 3, and 3, respectively. Each convolutional layer is followed by a ReLU activation and an adaptive average pooling layer. The pooled outputs from the three layers are concatenated and fed into a fully connected layer to produce predictions for the 52 gesture classes. Unlike FMC-DBNet, all parameters in the convolutional and fully connected layers are optimized through standard backpropagation.

To maintain strict fairness, the baseline model adopts the same training–test protocol described in Section 3.1, where repetitions {2, 5, 10} serve as the test set and the remaining repetitions are used for training. During training, 20% of the training portion is further split as a validation set for monitoring convergence and applying early stopping. This internal split does not alter the designated test set and therefore preserves full comparability with FMC-DBNet.

Training is performed using the Adam optimizer (learning rate of 1 × 10^−3^) with cross-entropy loss for a maximum of 50 epochs. The batch sizes are set to 64 and 32 for training and validation, respectively. To ensure reproducibility, the entire training procedure is repeated five times using random seeds {42, 123, 777, 2024, 2025}.

This baseline establishes the performance upper bound of a fully trainable CNN under the same data preprocessing and input constraints. Its results are reported in Section 3.6 alongside FMC-DBNet to enable a fair quantitative comparison in terms of accuracy, training cost, and model efficiency.

#### 3.5.2. ROCKET-Based Random Convolution Baseline

In addition to the trainable CNN baseline, a ROCKET-based model was included as a lightweight, training-free baseline for time-series classification. ROCKET employs a large set of randomly initialized and fixed one-dimensional convolutional kernels, followed by simple statistical feature extraction and a linear classifier, without using backpropagation.

In this work, ROCKET is applied only to the raw sEMG signals (without IMF information) after GLR-based segmentation and temporal normalization to a fixed length of 1100 samples. A total of 5000 random convolutional kernels are generated, with kernel lengths randomly sampled from {7, 9, 11}. Convolutional weights are drawn from a zero-mean Gaussian distribution and mean-centered, while bias terms are uniformly sampled. Dilation factors and zero-padding are randomly assigned following the original ROCKET formulation. All kernel generation and feature extraction procedures strictly follow the original ROCKET design.

For each kernel and each input channel, two features are extracted: the maximum convolution response (MAX) and the proportion of positive values (PPV). These features are concatenated and classified using a Ridge classifier, with the regularization parameter selected via cross-validation on the training set using a logarithmically spaced candidate set.

To ensure fair comparison, the ROCKET baseline follows the same repetition-based training–test protocol as FMC-DBNet, where repetitions {2, 5, 10} are used for testing. During cross-validation, a repetition-wise GroupKFold strategy is adopted to prevent information leakage between correlated repetitions.

### 3.6. Model Efficiency and Comparative Analysis

#### 3.6.1. Comparison with CNN and ROCKET

Under the subject-dependent evaluation protocol, FMC-DBNet was compared with two representative baselines: the fully trainable single-branch 1D-CNN described in Section 3.5 and a ROCKET-based random convolution classifier implemented under the same data setting. All methods followed the identical preprocessing pipeline and the fixed subject-wise train/test splits specified in Section 3.1, so that the observed performance differences were attributable to model design under a consistent evaluation protocol.

**Figure 9:**
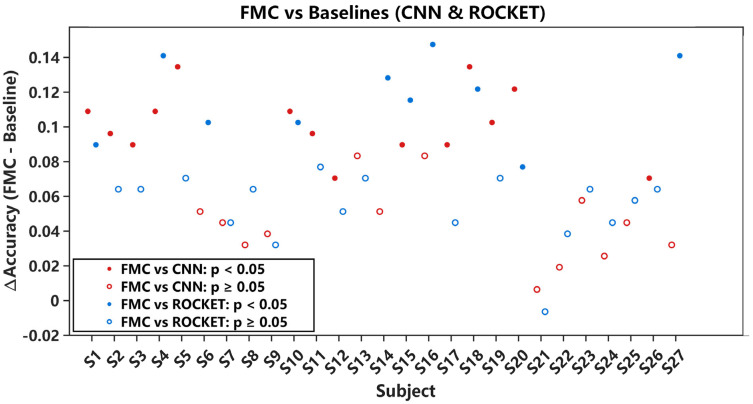
Subject-wise accuracy differences between FMC-DBNet and baseline models (CNN and ROCKET). Each marker corresponds to one subject. Solid markers indicate statistically significant differences based on McNemar’s test after Holm–Bonferroni correction (Holm-adjusted *p* < 0.05), whereas hollow markers denote non-significant differences.

In addition to average accuracy, subject-level statistical analysis was performed using McNemar’s test on paired test-set predictions. For each subject, FMC-DBNet and the corresponding baseline were evaluated on exactly the same test samples, forming a paired 2 × 2 contingency table from which exact two-sided McNemar *p*-values were computed. To control the family-wise error rate across the 27 subject-wise comparisons, Holm–Bonferroni adjustment was applied separately for the FMC-DBNet vs. CNN and FMC-DBNet vs. ROCKET comparisons. A subject-level difference was considered statistically significant when the Holm-adjusted *p*-value was below 0.05. Figure 9 shows the subject-wise accuracy differences (ΔAccuracy = ${Accuracy}_{FMC}$ − ${Accuracy}_{Baseline}$).

Solid markers indicate subjects with statistically significant differences after Holm–Bonferroni adjustment, whereas hollow markers denote non-significant differences. FMC-DBNet shows higher recognition accuracy than the trainable CNN for most subjects, and a substantial portion of these improvements remains significant after adjustment. Compared with ROCKET, FMC-DBNet also achieves higher accuracy on the majority of subjects, while fewer differences reach statistical significance, indicating the strong competitiveness of random convolution–based baselines under the same protocol.

For the McNemar analysis, predictions were aggregated across multiple runs with different random seeds into a single final decision for each test sample, enabling a one-to-one paired comparison at the sample level.

As a supplementary comparison, we summarize the recognition accuracy and computational cost of FMC-DBNet and the two baselines under the same evaluation protocol. Training time is defined as the total time for feature extraction and classifier fitting per subject, while single-sample inference time denotes the average prediction time per test sample.

The trainable CNN baseline achieved an average accuracy of 87.01% ± 4.30%, and the ROCKET baseline reached 88.66% ± 4.80%. In contrast, FMC-DBNet attained higher accuracy (e.g., 96.40% under the default setting) with substantially lower training overhead due to its frozen-convolution design.

Regarding computational cost, timing results are reported under standard execution settings and an additional CPU-only setting. Under standard settings, ROCKET (CPU) required 52.02 ± 0.38 s for training and 137.4 ± 0.7 ms/sample for inference; the trainable CNN (GPU) required 14.39 ± 0.11 s and 0.344 ± 0.07 ms/sample; and FMC-DBNet (GPU feature extraction + CPU Ridge) required 1.30 ± 0.01 s and 0.655 ± 0.077 ms/sample. Under CPU-only execution, FMC-DBNet required 8.53 s for training and 9.35 ms/sample for inference, whereas the trainable CNN required 155.49 s and 3.368 ms/sample, respectively.

Overall, these results indicate that FMC-DBNet provides a favorable accuracy–efficiency trade-off by achieving high recognition performance with markedly reduced computational cost while avoiding backpropagation-intensive optimization.

#### 3.6.2. Internal Analysis of FMC-DBNet

To assess the accuracy–efficiency trade-off, FMC-DBNet was evaluated with channel widths C∈{64, 128, 256, 512}. For each setting, 200 rounds of TPE-based hyperparameter search were conducted. The convolutional layers were randomly initialized and kept frozen, and an $L_{2}$-regularized Ridge classifier was used. Data partitioning followed Section 3.1.

**Figure 10:**
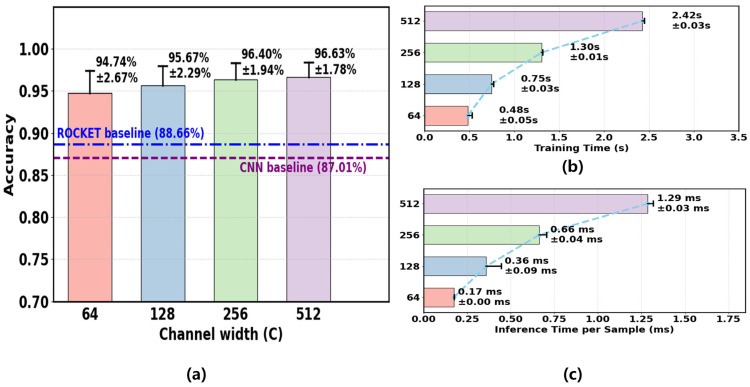
Performance and efficiency analysis of FMC-DBNet under different channel widths. (**a**) Mean test accuracy (±standard deviation) across 27 subjects, with the accuracies of the trainable CNN and ROCKET baselines shown for reference. (**b**) Training time of FMC-DBNet under different channel widths. (**c**) Single-sample inference time of FMC-DBNet under different channel widths.

As shown in Figure 10a, accuracy increased with channel width and saturated at C≥256. The mean test accuracy reached 96.63% at C=512, while C=64 already achieved 94.74%. Figure 10b,c report the corresponding training time and per-sample inference latency, both of which increased with model capacity. The training time in Figure 10b corresponds to the total training time per subject on the entire training set, rather than the time of a single batch.

**Figure 11:**
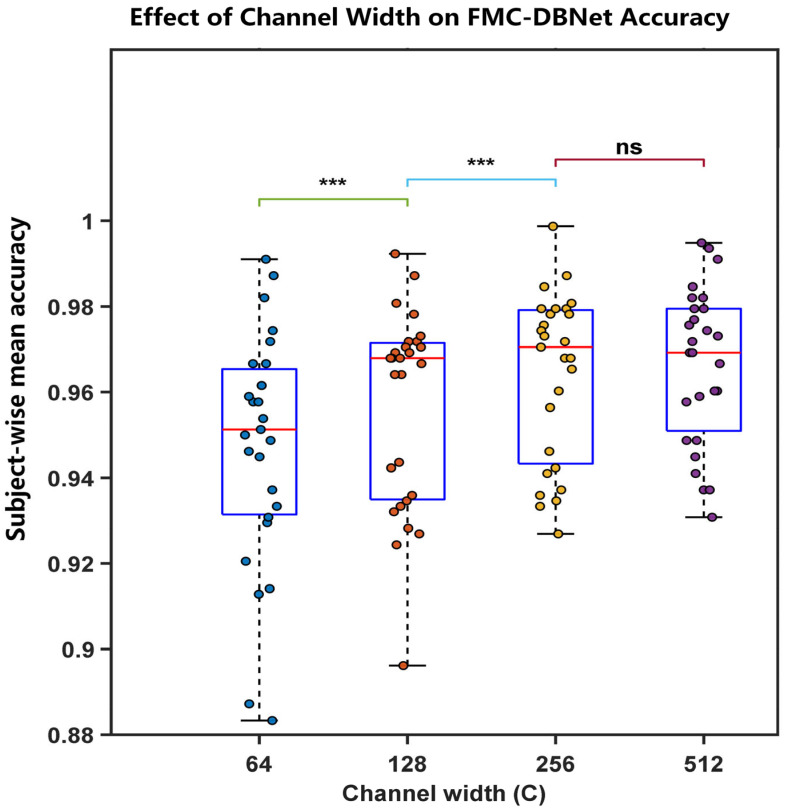
Subject-wise accuracy distribution and statistical significance analysis under different channel widths. Boxplots are constructed from the mean test accuracy of each subject averaged over random seeds, and scatter points indicate individual subjects. Overall differences are evaluated using the Friedman test, followed by pairwise Wilcoxon signed-rank tests with Holm–Bonferroni correction (^∗^ *p* < 0.05, ^∗∗^ *p* < 0.01, ^∗∗∗^ *p* < 0.001; ns: not significant).

A subject-wise nonparametric analysis (Figure 11) confirmed a significant overall effect of channel width (Friedman test, $p<10^{-9}$). Holm-corrected Wilcoxon tests showed significant gains up to C=256, whereas the difference between C=256 and C=512 was not significant. Therefore, C=256 was selected as the default configuration for subsequent experiments.

Under C=256, FMC-DBNet achieved an average accuracy of 96.40%, with a precision of 97.25%, a recall of 96.40%, an F1-score of 96.22%, and an MCC of 96.35%.

#### 3.6.3. Sensitivity Analysis of VMD Parameters

To examine the robustness of the proposed method with respect to VMD parameter selection, a sensitivity analysis was conducted on two key parameters: the number of modes K and the bandwidth penalty factor α. These parameters directly affect the decomposition characteristics of the extracted intrinsic mode functions (IMFs).

In this study, K is varied from 2 to 4, and α is selected from $\left\{500,\;1000,\;2000\right\}$. All other settings, including preprocessing, IMF selection strategy, network architecture, and classifier configuration, are kept identical to ensure a fair comparison. The same subject-wise training and testing protocol described in Section 3.1 is adopted.

The results, summarized in Table 4, indicate that the recognition performance remains generally stable across different parameter combinations. Increasing K beyond 2 does not lead to further performance improvement and instead introduces slight accuracy degradation, which is likely caused by redundant or weakly informative IMFs. Regarding α, smaller values may yield marginally higher accuracy in some cases; however, the performance differences across different α settings are minor.

Considering both classification performance and decomposition stability, K=2 and α=2000 are selected as a robust and well-balanced configuration. Overall, the proposed framework demonstrates low sensitivity to VMD parameter variations, confirming the robustness of the adopted settings.

### 3.7. Ablation Study

To assess the contribution of each component in FMC-DBNet, we conducted a series of ablation experiments under a consistent data split and evaluation protocol. The results are summarized in Table 5.

First, we compared GAP, PPV, and their combination as feature types. The results show that GAP + PPV achieves the highest accuracy (96.40%), outperforming the use of GAP alone (94.79%) or PPV alone (95.66%). This indicates that energy statistics and activation sparsity offer complementary temporal characteristics.

Second, regarding the input branches, the dual-branch configuration (Raw + IMF) outperforms either single-branch model, confirming the value of IMF components in providing complementary frequency-domain information and enhancing noise suppression.

In terms of convolutional structure, three configurations were evaluated: single-kernel convolution, multi-kernel without dilation, and multi-kernel with dilation. The accuracy increased from 94.37% with a single kernel to 95.92% with multiple kernels, and further to 96.40% after adding dilation. This indicates that multi-scale convolutions and dilated mechanisms better model both short- and long-term temporal dependencies.

Finally, in terms of the classifier, Ridge regression on frozen features clearly outperforms the trainable FC + Softmax approach (96.40% vs. 91.71%). This suggests that a linear classifier better preserves the stability of random frozen features, whereas end-to-end Softmax training tends to undermine their robustness.

In addition to the ablation results, we examined whether the GAP and PPV feature groups are utilized by the final linear classifier. Using the learned Ridge coefficients, the mean absolute weight is 0.00417 ± 0.00045 for GAP and 0.00708 ± 0.00071 for PPV, aggregated across subjects and random seeds. Both feature groups show consistently non-zero and stable weight magnitudes, suggesting that GAP and PPV jointly contribute to gesture discrimination, with PPV having a higher average contribution. This lightweight analysis provides quantitative evidence for the usefulness of the proposed statistical features without changing the overall model design.

### 3.8. Comparison with State-of-the-Art Methods

To further assess the effectiveness of the proposed FMC-DBNet, we compare it with several representative NinaPro DB1–based approaches reported in recent years. Table 6 summarizes the recognition performance of the compared models on the DB1 dataset.

All competing methods employ the same 52 gesture classes, the same set of 27 subjects, and comparable data-splitting protocols to ensure a fair evaluation. The comparison covers a diverse range of deep architectures, including the feature-enhanced PFNet, the multi-view fusion model HVPN, the multimodal framework sEMG-XCM, the multi-scale fusion network MCMP-Net, and the spatiotemporal hybrid model STMS-Net.

PFNet and HVPN achieve accuracies of approximately 87–88%, while sEMG-XCM and MCMP-Net reach around 91%. In contrast, the proposed FMC-DBNet attains 96.40% under the same experimental settings, substantially outperforming these representative approaches. This result indicates that, even with fully frozen convolutional kernels and no backpropagation, the multi-scale dual-branch architecture can still effectively capture stable discriminative temporal patterns, yielding superior accuracy and computational efficiency.

It is worth noting that minor differences exist in the experimental splits adopted by different studies, and we have aligned the settings as closely as possible to ensure fair comparison. Overall, the results show that FMC-DBNet offers a markedly better balance between accuracy and computational efficiency than most trainable deep models, demonstrating stronger practical value for small-sample sEMG recognition tasks.

## 4. Discussion

The proposed lightweight Frozen Multi-Convolution Dual-Branch Network (FMC-DBNet) achieves an average accuracy of 96.4% with a training time of only 1.30 s on the Ninapro DB1 dataset, substantially outperforming the conventional trainable CNN (87.0%), a ROCKET-based random convolution baseline, and several hybrid architectures [22,24]. Across 27 subjects and multiple random trials, the model maintains consistently stable performance, indicating that the frozen convolutional structure preserves strong cross-subject generalization despite its extremely low computational cost.

From the perspective of feature learning, the dual-branch design explicitly decouples raw sEMG signals from their VMD-derived IMFs, enabling complementary modeling of high-frequency muscular activation patterns and low-frequency modal trends. The time–frequency locality of VMD suppresses non-stationary components, and in combination with the multi-criteria IMF selection strategy [41], provides cleaner and more interpretable time–frequency representations without increasing model complexity. This improved representation contributes directly to the network’s robustness against noise and inter-subject variability.

In terms of convolutional feature extraction, the model employs randomly initialized and frozen multi-scale kernels, following the paradigm of random feature mapping used in RVFL [27,28,29], ROCKET [30], and edRand-CNN [34]. The statistical aggregation of PPV and GAP further compensates for the absence of trainable kernels, enabling the network to capture both localized activation patterns and global energy structures under fixed weights. The experimental results confirm that this “frozen convolution + statistical aggregation” strategy can still produce highly discriminative features without relying on backpropagation.

In terms of efficiency, FMC-DBNet requires only 1.30 s for training, making it roughly ten times faster than a trainable CNN. Together with the findings of Saxe et al. [46] and Ovadia et al. on the stability of random-weight networks, our results further demonstrate that random convolutional architectures exhibit strong generalization and robustness to distributional shifts in sEMG recognition tasks.

Despite its strong performance, this study has several limitations. The model relies on VMD hyperparameters and the IMF selection strategy, which may be sensitive to signal quality variations across different data sources. In addition, the current evaluation is restricted to Ninapro DB1, and its cross-database and cross-device generalizability remains unverified. Future work may explore adaptive mode decomposition, end-to-end decomposition networks, and further validation on datasets such as DB2, DB5, and CapgMyo. Integrating transfer learning or incremental learning strategies may also enhance the model’s adaptability in real-world, complex environments.

In summary, FMC-DBNet achieves efficient, lightweight, and interpretable sEMG feature modeling through temporal–spectral decoupling, multi-scale random convolutions, and statistical feature aggregation, offering a promising solution for low-power wearable devices and real-time myoelectric control systems.

## 5. Conclusions

This study proposes a lightweight Frozen Multi-Convolution Dual-Branch Network (FMC-DBNet) for sEMG-based gesture recognition. The method extracts temporal–spectral features from both raw signals and IMFs via VMD and aggregates them through PPV and GAP without any trainable parameters. Without relying on backpropagation, the model achieves an average accuracy of 96.4% on Ninapro DB1 while reducing training time by approximately 90% compared with a trainable CNN, demonstrating the effectiveness and computational advantages of frozen random convolutions for myoelectric recognition.

From a theoretical perspective, FMC-DBNet extends random feature learning into the convolutional domain by integrating the random mapping principle of RVFL with the random convolution mechanisms of ROCKET and edRand-CNN, while incorporating a VMD-based physiological feature pathway. This design remains lightweight while offering both interpretability and robustness.

Future research may proceed along three directions. First, exploring adaptive or end-to-end mode decomposition approaches to reduce reliance on VMD hyperparameters. Second, evaluating cross-subject and cross-device generalization on datasets such as DB2, DB5, and CapgMyo. Third, deploying the model on low-power hardware platforms to support real-time applications in rehabilitation aids and wearable devices.

Overall, FMC-DBNet provides a new direction for applying random convolutions to physiological signal recognition and achieves a well-balanced trade-off among performance, efficiency, and deployability.

## Figures and Tables

**Table 1:** Evaluation Metrics and Their Mathematical Formulations for Classification Performance.

Metrics	Formula
Accuracy	$ACC=\left(\frac{TP+TN}{TP+FN+TN+FP}\right)\times 100$
Sensitivity	$SEN=\left(\frac{TP}{TP+FN}\right)\times 100$
Precision	$PRC=\left(\frac{TP}{TP+FP}\right)\times 100$
F1-Score	$F1\text{-}S=2\times \left(\frac{PRC\times SEN}{PRC+SEN}\right)\times 100$
Matthews Correlation Coefficient	$MCC=\left(\frac{\left(TP\times TN\right)-(FP\times FN)}{\sqrt{\left(TP+FP)(TP+FN)(TN+FP)(TN+FN\right)}}\right)\times 100$

**Table 2:** Subject S1—Gesture 5, Repetition 8, Channel 1.

IMF	PCC	Energy	Entropy	Score
IMF1	0.987	0.972	0.543	0.892
IMF2	0.319	0.028	0.021	0.143

**Table 3:** Architecture and Layer-wise Configuration of the FMC-DBNet.

Layer	Type	Output Shape	Kernel(k,d)	No. of Filter	Activation	Features	Parameters
1	Conv1D (Raw)	(1100,256)	(3,1)	256	ReLU	GAP + PPV	7936 (frozen)
2	Conv1D (Raw)	(1100,256)	(5,3)	256	ReLU	GAP + PPV	13,056 (frozen)
3	Conv1D (Raw)	(1100,256)	(11,8)	256	ReLU	GAP + PPV	28,416 (frozen)
4	Conv1D (IMF)	(1100,256)	(3,1)	256	ReLU	GAP + PPV	7936 (frozen)
5	Conv1D (IMF)	(1100,256)	(5,3)	256	ReLU	GAP + PPV	13,056 (frozen)
6	Conv1D (IMF)	(1100,256)	(11,8)	256	ReLU	GAP + PPV	28,416 (frozen)
7	Raw branch fusion	(1,1536)	_	_	_	_	49,408
8	IMF branch fusion	(1,1536)	_	_	_	_	49,408
9	Branch fusion	(1,3072)	_	_	_	_	98,816
10	Ridge Classifier	(1,52)	_	_	_	_	159,744

**Table 4:** Test accuracy (%) under different VMD parameter settings (mean ± std across 27 subjects).

K	α = 500	α = 1000	α = 2000
2	96.45% ± 2.08%	96.41% ± 2.02%	96.38% ± 1.92%
3	96.45% ± 1.98%	96.28% ± 2.10%	96.31% ± 2.21%
4	96.35% ± 1.94%	96.25% ± 2.07%	96.34% ± 2.10%

**Table 5:** Comparison of the classification accuracy of our model and other methods.

No.	Model Variant	Feature Type	Input Branch	Convolution Structure	Classifier	Accuracy
1	GAP only	GAP	Raw + IMF	Multi-kernel	Ridge	94.79%
2	PPV only	PPV	Raw + IMF	Multi-kernel	Ridge	95.66%
3	GAP + PPV	GAP + PPV	Raw + IMF	Multi-kernel	Ridge	96.40%
4	Raw only	GAP + PPV	Raw	Multi-kernel	Ridge	96.01%
5	IMF only	GAP + PPV	IMF	Multi-kernel	Ridge	92.72%
6	Single-kernel (k = 5, d = 1)	GAP + PPV	Raw + IMF	Single kernel	Ridge	94.37%
7	Multi-kernel (d = 1)	GAP + PPV	Raw + IMF	Multi-kernel	Ridge	95.92%
8	Ridge with FC + Softmax	GAP + PPV	Raw + IMF	Multi-kernel	FC + Softmax	91.71%

**Table 6:** Comparison of different gesture recognition methods.

Reference	Labels	Subjects	Training Trials	Test Trials	Method	Accuracy
WentaoWei et al. [37] 2021	52	27	1, 3, 4, 6, 7, 8, 9	2, 5, 10	PFNet	87.60%
WentaoWei et al. [38] 2021	52	27	1, 3, 4, 6, 7, 8, 9	2, 5, 10	HVPN	88.40%
Qingfeng Dai et al. [39] 2023	52	27	1, 3, 4, 6, 7, 8, 9	2, 5, 10	sEMG-XCM	91.40%
Aly Medhat Moslhi et al. [47] 2024	52	27	1, 3, 4, 6, 8, 9, 10	2, 5, 7	ST-Nina-RAW	85.97%
Xiang Mian et al. [48] 2024	52	27	1, 3, 4, 6, 8, 9, 10	2, 5, 7	MCMP-Net	91.8%
WANG SIJIN et al. [49] 2025	52	27	1, 3, 4, 6, 8, 9, 10	2, 5, 7	STMS-Net	91.9%
Ours approach	52	52	1, 3, 4, 6, 7, 8, 9	2, 5, 10	FMC-DBNet	96.40%

## Data Availability

The publicly available dataset NinaPro DB1 was used in this study. The dataset can be accessed at http://ninapro.hevs.ch (accessed on 4 March 2025).

## Appendix A. Detailed Experimental Configuration

### Appendix A.1. Dataset and Subject-Dependent Evaluation Protocol

Experiments were conducted on the Ninapro DB1 dataset using recordings from 27 subjects (S1–S27). For each subject, data from the three exercise subsets (E1–E3) were utilized. A subject-dependent evaluation protocol was adopted throughout this study.

To ensure a fair and consistent comparison across all methods, identical preprocessing steps and subject-wise train/test splits were applied, as described in Section 3.1. All models were evaluated independently for each subject without cross-subject data mixing. After segmentation and boundary refinement, each EMG segment was temporally normalized to a fixed length of 1100 samples prior to feature extraction and classification.

### Appendix A.2. Signal Preparation and Low-Pass Filtering

For each subject, raw EMG recordings from the three exercise subsets were concatenated into a continuous signal sequence, together with the corresponding stimulus labels and repetition indices.

A first-order Butterworth low-pass filter with a cutoff frequency of 1 Hz was applied independently to each EMG channel. Filtering was implemented using a zero-phase forward–backward scheme to avoid phase distortion. The sampling frequency of the EMG signals was 100 Hz, consistent with the Ninapro DB1 acquisition protocol.

Filter configuration: Butterworth low-pass filter (order = 1), cutoff frequency = 1 Hz, sampling rate = 100 Hz, zero-phase implementation.

### Appendix A.3. GLR-Based Gesture Boundary Refinement

Gesture boundaries were refined using a generalized likelihood ratio (GLR) framework under a two-state hypothesis model corresponding to rest and motion states.

For each labeled gesture repetition, an expanded temporal window was constructed by extending the initial annotated segment by 300 samples on both sides. Within this window, the rectified EMG signal of each channel was analyzed to determine the onset and offset points that maximize the log-likelihood of a two-segment Gaussian model, subject to a minimum duration constraint.

To enhance robustness against channel-specific noise and variability, a multi-channel fusion strategy was employed. The final onset was defined as the minimum onset detected across valid channels, while the final offset was defined as the maximum offset. A gesture segment was accepted only when a sufficient number of channels yielded consistent onset–offset estimates and the resulting segment length exceeded the predefined minimum duration.

GLR configuration: extension length = 300 samples, minimum duration = 50 samples, search stride = 3 samples, multi-channel fusion rule (onset = minimum, offset = maximum), minimum valid-channel requirement = 3.

### Appendix A.4. VMD Configuration and IMF Extraction

For each refined gesture segment, variational mode decomposition (VMD) was applied independently to each EMG channel to extract intrinsic mode functions (IMFs). The decomposition was performed without enforcing a DC mode and with a fixed internal convergence tolerance.

To evaluate the robustness of the proposed framework with respect to VMD parameter selection, a sensitivity analysis was conducted over the parameter grid K∈{2, 3, 4} and α∈{500, 1000, 2000}, while all other settings, including preprocessing, IMF selection strategy, network architecture, and classifier configuration, were kept unchanged.

VMD configuration: τ=0, DC = 0, initialization scheme = 1, convergence tolerance = $10^{-7}$; sensitivity grid K∈{2, 3, 4}, α∈{500, 1000, 2000}.

### Appendix A.5. Weighted IMF Selection (Top-1 Strategy)

To identify informative IMF components for the dual-branch representation, a weighted scoring criterion was employed to rank IMFs for each channel.

For the k-th IMF of a given channel, three indicators were computed:

(i) The absolute Pearson correlation with the corresponding raw EMG signal,
(ii) A normalized energy ratio,
(iii) Shannon entropy.

Each indicator was min–max normalized on a per-channel basis. The final score was computed as:

(A1) $$Score_{i}=\omega_{p}{\rho^{\prime }}_{i}+\omega_{e}E_{i}+\omega_{s}(1-{S^{\prime }}_{i}),$$

where $w_{p}=0.4$, $w_{e}=0.4$, and $w_{s}=0.2$. For each channel, the IMF with the highest score (Top-1) was selected and used for subsequent processing.

IMF selection settings: Top-1 IMF per channel; weights = (0.4, 0.4, 0.2); entropy type = Shannon; per-channel min–max normalization.

### Appendix A.6. Feature Extraction with Frozen Multi-Kernel CNN (FMC-DBNet)

The proposed FMC-DBNet adopts a dual-branch architecture. One branch processes the normalized raw EMG segments, while the other branch processes the corresponding IMF-based representations.

Each branch consists of frozen (non-trainable) one-dimensional convolutional layers with multiple kernel sizes to capture multi-scale temporal patterns. In the main configuration, three kernel sizes (3, 5, 11) with corresponding dilation rates (1, 3, 8) were employed. Convolutional weights were randomly initialized using Kaiming initialization and kept fixed throughout all experiments.

For each convolutional feature map, two lightweight statistical descriptors were extracted: global average pooling (GAP) and positive proportion (PPV), defined as the proportion of positive activations along the temporal dimension. Features from multiple kernels were concatenated within each branch, and the two branches were fused by concatenation to form the final feature representation.

CNN configuration: multi-kernel 1D convolution; kernel sizes = (3, 5, 11); dilations = (1, 3, 8); frozen random weights (Kaiming initialization); feature statistics = GAP + PPV; branch fusion = concatenation.

### Appendix A.7. Classification and Timing Definition

Classification was performed using an L2-regularized Ridge regression model with a fixed regularization coefficient α=0.039. For each subject, experiments were repeated using five different random seeds (42, 123, 777, 2024, 2025).

Five-fold cross-validation was conducted on the training set to report cross-validation accuracy. Training time was defined as the total time required for (i) frozen CNN feature extraction on the training set and (ii) Ridge model fitting. Test time was defined as the total time required for (i) frozen CNN feature extraction on the test set and (ii) Ridge prediction. The single-sample inference time was computed by dividing the total test time by the number of test samples.
